# To the Editors of the Medical and Physical Journal

**Published:** 1801-07

**Authors:** Thomas Peck

**Affiliations:** Higham Ferrers


### *To the Editors of the Medical and Physical Journal.*

GENTLEMEN,

AMONG the incalculable and rapid improvements of the past century, mankind have, perhaps, in *no instance* been more benefited than by the advances which have been made in the *healing* art: And though the progression has been truly great, there are, yet, no reasons to conclude we have reached the acme of knowledge, or arrived at the ne plus ultra of medical science. There is more to accomplish; and it behoves every practitioner to promote, by all means in his power, the furtherance of useful information.

> Qui studet optatam cursu contingere Metam,

> Multa tulit fecitque puer.—— HORACE.

Though our predecessors have done much towards the relief of suffering humanity, yet has it been left for the present period to glory in *very* important discoveries, and make its boast of characters, whose unceasing endeavours have been crowned with copious success, whose active exertions have been emblazoned with the most brilliant lustre. Witness the indisputable extermination of that dire scourge of nations, the Small-pox, by whose destructive ravages thousands have been impetuously precipitated to "that bourne from whence no traveller returns." A Jenner has been ordered to arrest its insatiate rage, and instrumentally to save mankind from a devouring malady by a benign and effectual agent, whose effects on the system are comparitively trifling, and which bears with it this important recommendation, *'Tis not contagious.**

Improvements in surgery deserve our regard, and particularly simplicity in operations, whereby the sufferings of the patient are lessened, while the operator is not subject to embarrasment by that farrago of instruments used by ancient surgeons. I would, here, just glance at the decided preference of puncturing in the linea alba with an imposthume lancet, to the very crude method of violently (and always uncertainly) thrusting through the parietes an uncourtly trocar, in paracentesis. I have twice performed it in my own practice, and have witnessed it in seven instances on one patient in this neighbourhood, where I had the honour of being called to assist a late senior practitioner

* The profession should do all in their power to remove from the minds of their acquaintance, that prejudice which is certain, *for some* time, to operate, against innovations.

practitioner at Wellingbrough. In none of the cases was there the smallest difficulty, or the least prevention, to union by the first intention; while the pain given by the lancet was very inconsiderable.

Great also have been the improvements in the management of foul asthenic ulcers, which have frequently baffled many practitioners, and been seriously offensive, *as* well as exquisitely painful, to the subjects of them. I observe, in your last Journal (page 562), Dr. Stutz's ideas relative to their cure by the caustic alkali; and I am happy in being able to add to the stock of information on that subject. I have in many cases used the following solution with invariable success: ℞. Argent. nitrat. gr. ij. aq. ferventis ℥j. tinct. opii. ʒij. ft. solut. The ulcers are covered with lint made wet with this solution, and a bandage of thin flannel constantly worn. In three or four days a disposition to sloughing appears, and the cure will be effected by the ointment as follows: ℞. Hydrarg. nit. rub. ʒj. pulv. opii. ʒss. tere simul & misce sedulo cum ung. res. flav. ℥i. ft. ung. By means of this ointment *alone* I have cured several ulcers of this nature; first, however, removing their indurated edges with a scalpel; for there will be no disposition to healthy granulation during *their* existence. The very *inert* nature of those ulcers must have been frequently noticed by every practitioner, and nothing can be more rational than to attempt their cure on the principle of destroying such sluggish tendency, by inducing new action by brisk *stimuli*, the *preferable* of which, in my opinion, is the solution above recommended.

Two cases of a very inveterate kind have lately come under my care; but in *none* have I had occasion to use a stronger solution, or to adopt a different mode of application.

I am, &c.

